# Carbon monoxide oxidizers in soils of different ages from Piton de la Fournaise volcano

**DOI:** 10.1093/femsec/fiag062

**Published:** 2026-06-09

**Authors:** Constance Wildbur, Robin A Dawson, Shamik Roy, Claudine Ah-Peng, Mikk Espenberg, Marcela Hernández

**Affiliations:** School of Biological Sciences, University of East Anglia, Norwich NR4 7TJ, United Kingdom; School of Biological Sciences, University of East Anglia, Norwich NR4 7TJ, United Kingdom; School of Biological Sciences, University of East Anglia, Norwich NR4 7TJ, United Kingdom; Chair for Forest Zoology, Technische Universität Dresden, Tharandt 01737, Germany; UMR PVBMT, Université de la Réunion, Saint-Pierre 97410, La Réunion, France; OSU-Réunion, Université de la Réunion, CNRS, IRD, Météo France, Saint-Denis 97400, La Réunion, France; Department of Geography, Institute of Ecology & Earth Sciences, University of Tartu, Tartu 51013, Estonia; School of Biological Sciences, University of East Anglia, Norwich NR4 7TJ, United Kingdom

**Keywords:** carbon monoxide dehydrogenase, *cox* genes, metagenome-assembled genome, soil microbes, volcano

## Abstract

Volcanic soils provide a unique environment for studying microbial colonization and succession due to their extreme conditions and distinct geochemical profiles. This study focused on carbon monoxide (CO)-oxidizing microbial communities in volcanic soils at Piton De La Fournaise, Réunion Island. Soil samples from three sites (corresponding to eruptions in 1401, 1559, and 2007) were analysed to assess microbial community structure using 16S rRNA gene sequencing and metagenomic analysis to identify functional genes involved in CO oxidation. Phylum-level analysis showed higher relative abundance of Acidobacteriota and Chloroflexota, lower abundances of Actinomycetota and Bacteroidota, and relatively stable levels of Pseudomonadota, while class-level patterns included rising Alphaproteobacteria and Acidobacteriia, with Ktenobacteria emerging in the 1401 site. CO dehydrogenase-related genes were found in 17 metagenome-assembled genomes across all sites. The CO consumption rate by microbes in soils was measured. CO-oxidizing microbes were present across soil ages, with detectable activity in the 2007 site and greatest activity in the 1401 site, suggesting that these microbes actively use CO as an energy source even in soils with primary vegetation, contrary to general understanding. The findings suggest intricate dynamics of microbial succession in volcanic soils and may challenge conventional expectations about community complexity over time.

## Teaser

Volcanic soils host diverse CO (carbon monoxide)-oxidizing microbes whose composition and activity shift with soil age. Genes and activity occurred across all sites, with highest CO oxidation in oldest soils, revealing unexpected succession dynamics.

## Introduction

Volcanic sites provide a model for analyzing how soil is formed through microbial interactions starting off with important pioneer microbes that are capable of surviving in extreme environments (Wubs et al. 2016, Fantom et al. 2025). The young volcanic soils can be classified as extreme environments due to the very low amount of organic matter (King 2003a, Fujimura et al. 2011). These pioneer microbes display physiological and functional traits, which significantly influence nutrient cycling, thereby allowing soil succession to occur (King 2003a). Understanding how these pioneer microbes can inhabit these sites and influence succession helps us to understand more about effective soil restoration, which is becoming more important due to the anthropogenic degradation of the environment. The lack of organic matter in recent volcanic deposits forces the use of alternative energy and carbon sources. It is known that volcanoes produce trace amounts of reduced gases such as carbon dioxide (Di Muro et al. 2016), carbon monoxide (CO), hydrogen sulfide, hydrogen, and methane. Through metagenomic functional analysis on soil samples collected from various volcanoes worldwide, genes that allow microorganisms to use CO as carbon and energy sources have been identified (Shepherd 1925, Robb and Techtmann 2018, Hernández et al. 2020a). Therefore, the development rate of these ecosystems could depend on the microbial utilization of trace gases as energy sources (King 2003a).

Carbon monoxide dehydrogenase (CODH) is a key enzyme in catalyzing the oxidation of CO in microorganisms. CODH is a multi-subunit enzyme encoded by *coxMSL*, with the Mo–Cu-containing active site found in CoxL (Bährle et al. 2023). *coxMSL* have been identified in bacteria from the phylum Acidobacteriota, Actinomycetota, Chloroflexota, Pseudomonadota, and Bacillota (Schübel et al. 1995, Dunfield and King 2004, Hernández et al. 2020a, Dawson et al. 2025), reflecting the ecological adaptation of CO-oxidizing microorganisms to different environments.

This study investigates the pioneer microbial communities and how they vary across volcanic soils of different ages on Réunion Island (Indian Ocean). For this study, we consider these sites as forming a putative chronosequence, representing different stages of ecosystem development following volcanic eruptions (1401, 1559, and 2007). These sites differ in age since formation and share broadly similar geological context, while environmental conditions also influence soil development. Réunion Island is a basaltic volcanic edifice comprising two main volcanic systems: the inactive Piton des Neiges, built between ∼2.2 and 0.027 Ma, and active Piton de la Fournaise, located in the southeastern part of the island, which was developed since ∼0.56 Ma (O’Hara et al. 2026). Piton de la Fournaise is one of the most active volcanoes in the world (Morandi et al. 2016). The climate of Piton de la Fournaise is humid and tropical, with an annual mean temperature range from 18°C to 25°C and the driest months of precipitation between 75 and 400 mm (Jumeaux et al. 2011). Therefore, the lower part of Piton de la Fournaise is covered by tropical rainforests (Strasberg 1994). Past lava flows have affected upper elevations down to sea level. Any flows <1000 years old have been observed to have very thin topsoil (Meunier et al. 2010). Newly exposed volcanic deposits harbour diverse microbial communities despite harsh conditions such as low pH and minimal organic matter, comparable to other extreme terrestrial environments (Gomez-Alvarez et al. 2007).

Notable patterns emerged when investigating microbial succession on volcanic deposits, highlighting the critical role of suitable substrates for energy and biosynthetic metabolism, with high microbial abundance and diversity influenced by environmental gradients such as increased organic carbon and vegetation presence (King 2003a, Weber and King 2010a). At Llaima Volcano in Chile, Chloroflexota, Verrucomicrobiata, and Planctomycetota were found to dominate the younger soils, while Pseudomonadota and Acidobacteriota prevailed in the older sites (Hernández et al. 2020b). The microbial populations in particularly vegetated, young soils exhibited metabolic versatility, as they contained the genetic potential to oxidize CO, hydrogen, or methane as energy sources, indicating facultative chemolithoautotrophic, or mixotrophic capabilities (Hernández et al. 2020a).

Studies conducted on CO oxidizers within volcanic environments offer valuable insights into their widespread presence and role in contributing to the development of complex microbial communities during soil formation. For example, *coxL* genes were identified in metagenome-assembled genomes (MAGs) recovered from various soil ages at Llaima Volcano in Chile (Hernández et al. 2020a). Moreover, CO oxidizer abundance and diversity tend to increase with vegetation and organic matter concentrations (King 2003a, Weber and King 2010), similar to other microbes found in these communities, although the impact of CO oxidation may be negligible in vegetated environments (King and Weber 2008). Despite extreme conditions, volcanic deposits support a diverse microbial community (Guo et al. 2014, Byloos et al. 2018), with aerobic CO oxidation being documented across numerous bacterial lineages revealing novel functional capacities within these environments (Dunfield and King 2004, Dawson et al. 2025). Given that Piton de la Fournaise is an island volcano with strong elevational gradients, we aimed to investigate whether CO-oxidizing groups such as Pseudomonadota, Acidobacteriota, and Ktedonobacteria follow similar succession patterns to those reported from continental volcanic systems. We also sought to determine whether CO oxidation remains an active metabolic process in older soils, where vegetation and organic carbon inputs have become established. We hypothesized that microbial community complexity and abundance increase with soil age and that CO-oxidizing microbes would be predominantly active in younger soils, where organic matter is limited.

## Materials and methods

### Sample collection and physico-chemical properties determination

**Figure 1:**
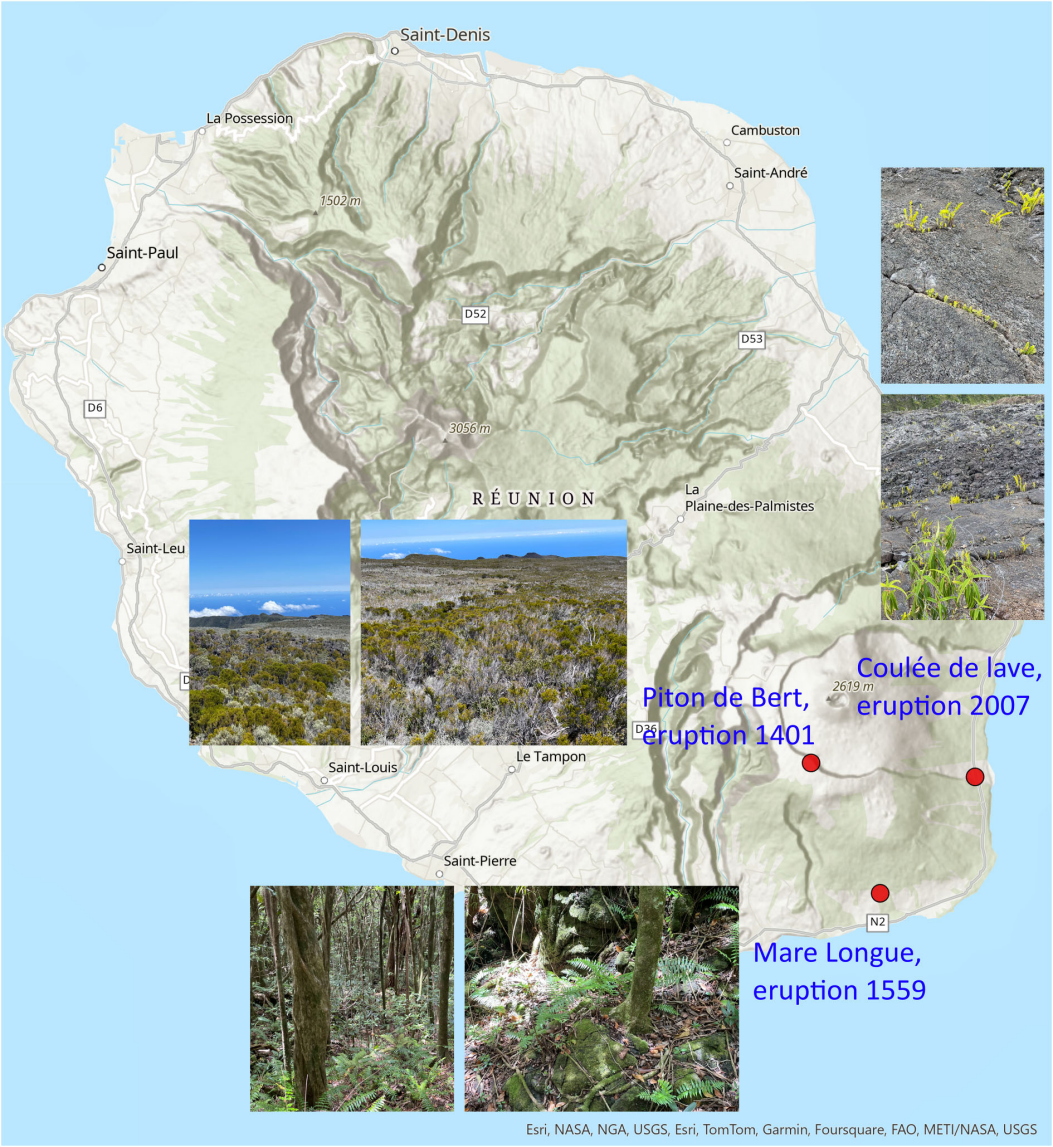
Map of the studied lava flows of Piton de la Fournaise volcano on Réunion Island.

Soils were sampled in November 2022 at three different locations of the volcano: Piton de Bert (PDB) (2242 masl, 21.2788831 S, 55.6980607 E), Mare Longue (ML) (282 masl, 21.3512651 S, 55.7392276 E), and Coulée de lave (CDL) (124 masl, 21.2866284 S, 55.7957900 E) (Fig. 1), which were formed by eruptions in 1401, 1559, and 2007, respectively (Albert et al. 2020). Soil samples from the CDL site were collected from the rock crevices. Three distinct vegetation types are represented at these three sites. PDB hosts subalpine vegetation composed of endemic shrubs such as *Erica reunionensis* E.G.H. Oliv., which are of small stature (<2 m). ML represents a rich native primary lowland forest with a canopy height of about 20 m. CDL hosts pioneer lowland vegetation, sparsely vegetated and composed of the lichen *Stereocaulon vulcanii* (Bory) Ach., two dominant moss species [*Campylopus aureonitens* (Müll. Hal.) A. Jaeger and *Polytrichum subpilosum* P. Beauv.], and the fern *Nephrolepis abrupta* (Bory) Mett. The CDL site may also contain some non-native plant species such as *Boehmeria penduliflora* Wedd. ex. D.G. Long, particularly in areas close to the road. Two cm of topsoil was removed at each site, and three random samples were taken in the pattern of a triangle for each site. Samples were shipped in Ziploc bags and then stored at 5°C in the laboratory. Air and soil temperatures were recorded *in situ* using a temperature logger (Comet Systems Ltd., Rožnov pod Radhoštem, Czech Republic). Measurements may vary due to local conditions including canopy cover, elevation, and cloud cover.

pH was measured by mixing soil samples with distilled water in a 1:10 ratio (w/v), shaking samples at 180 r/m for 5 min in an orbital shaker and allowing to rest for 2 h before measuring with a FiveEasy pH meter (Mettler Toledo). Soil moisture content was determined by recording the weight of the empty container and weight of soil before incubation, storing samples overnight in an oven at 100°C, and then recording dried soil weights. Soil moisture content was then calculated as a percentage (Table S1).

### CO uptake by soil microbes

For all samples, 12 g of soil was sifted to remove rocks and plant matter and added to a 120 ml serum vial. CO was added from a 5000 ppm (0.5% in N_2_) stock solution to a final concentration of ∼100 ppm, according to King and King (2014). CO concentrations were recorded using an Agilent Technologies 7890A Gas Chromatograph according to Dawson et al. (2025). Briefly, sample gases were separated using an HP-Molesieve PLOT column (Agilent). CO was converted to methane by hydrogenation reaction on a nickel catalyst (350°C) before detection by a flame ionization detector. Measurements were taken for >200 h, or until CO consumption was no longer detected. The rate of CO consumption was calculated from the initial rate of CO uptake in the vials, based on linear regression applied to the first five data points after CO uptake, corresponding to 30–150 h for both CDL and ML, and 10–50 h for PDB. Standards were prepared in N_2_-flushed 120 ml vials from 100 to 1 ppm CO. To ensure the activity in the observed vials was biological, control samples were made by autoclaving the soil for 60 min and leaving residual CO to diffuse out in a fume hood. The activity was measured by the same method indicated above (Fig. S1). The experiments were conducted in triplicate (biological replicates).

### DNA extraction from volcanic soils

0.25 g of soil was homogenized using a mortar and pestle, with cleaning using 70% (v/v) ethanol and distilled water between uses. DNA was extracted from homogenized soils using a DNeasy PowerSoil Pro Kit (Qiagen), with extracted DNA immediately cleaned using a PowerClean pro Cleanup Kit (Qiagen). DNA concentrations were calculated using a Qubit dsDNA HS Assay Kit (Thermo Fisher Scientific). DNA was stored at −20°C until sequencing. The same DNA extracts were used for bacterial community composition and metagenomic analyses.

### Bacterial community composition

The 16S rRNA gene was amplified using primers 341F (CCTAYGGGRBGCASCAG)/806R (GGACTACNNGGGTATCTAAT) from ≥200 ng total DNA from each soil sample and sequenced on an Illumina PE250 platform (NovaSeq 6000) at Novogene (Cambridge, UK). Library preparation was carried out using NEBNext® Ultra™ IIDNA Library Prep Kit (catalogue number E7645) at Novogene. Sequences were quality-filtered using QIIME, including removal of low-quality reads and trimming based on Phred quality scores, before downstream processing (Caporaso et al. 2010). For every sample, chimeras were removed using reference-based chimera checking with VSEARCH 2.16.0 (Rognes et al. 2016). The filtered reads wer e then clustered into operational taxonomic units (OTUs) at 97% similarity using UPARSE (Edgar 2013). Taxonomy was assigned using SILVA_v138 database (Quast et al. 2013).

### Statistical analyses and OTU classifications

Microbial alpha-diversity was expressed as species richness, Shannon index, species evenness (Pielou’s evenness index), and Simpson’s diversity. We used linear models to assess the effect of soil age on CO consumption rates and all four measures of alpha-diversity. Here, in separate models, we modelled alpha-diversity measures and CO consumption rates as dependent variable, and soil age (soils destroyed from the eruption at different times; 3-levels) as explanatory variable (see R script in the Supplementary informatio*n*). For beta-diversity, we visualized variation in microbial community composition using Principal Coordinates Analysis (PCoA) with the ‘pcoa’ function, based on Bray–Curtis dissimilarity of the normalized OTU dataset, transformed with the Hellinger transformation (Legendre and Gallagher 2001) using the ‘decostand’ function. We then used constrained ordination with Canonical Correspondence Analysis (CCA) to assess whether the observed variability in the microbial communities is explained by soil age. All analyses were performed in R 4.3.2 using nlme, vegan, and ape packages (see R script in the Supplementary information).

Statistically significant differences in CO consumption rates under the different conditions were assessed in triplicate using one-way analysis of variance (ANOVA) followed by a Tukey *post hoc* test using R studio using the vegan package.

### Metagenomic functional analysis

Total DNA (≥200 ng) for metagenomic analysis was also sequenced by Novogene. The genomic DNA was randomly sheared, end repaired, A-tailed, and ligated to Illumina adapters. After ligation, the fragments were size selected, then PCR amplified using the universal Illumina adapter primers to enrich adapter ligated molecules and finally purified to remove leftover reagents and small fragments. Library preparation was conducted by Novogene following their standard protocol. Library preparation was carried out using NGS DNA Library Prep Set (catalogue number PT004). Raw reads were trimmed at Novogene using fastp (version 0.23.1) with the following parameters: *-g -q 5 -u 50 -n 15 -l 150 –overlap_diff_limit 1 –overlap_diff_%_limit 10*. This step was used to remove reads containing adapters, reads containing ambiguous bases (N), and low-quality reads. FastQC (version 0.11.9) was employed to assess the quality of raw sequencing data obtained from the samples (Andrews 2010). Metagenomic assembly was performed to reconstruct the microbial genomes present in the environmental samples from the sequenced reads using SPAdes (version 3.14.0; Bankevich et al. 2012, Nurk et al. 2017) with Phred+33 quality score encoding (–phred-offset 33) and a k-mer series of 21, 33, and 55. Binning analysis was performed with scaffolds larger than 1000 bp. MetaWRAP (version 1.2.1) was used for binning analysis, leveraging three binning software programs: MaxBin2, MetaBAT2, and CONCOCT (Alneberg et al. 2014, Kang et al. 2015, Wu et al. 2016, Uritskiy et al. 2018). The resulting bins were further refined using the bin_refinement module within MetaWRAP, retaining only those with completeness >60% and contamination <10%. Identification was carried out using GTBDK database (version 2.5.2, GTDB-Tk release R226) (Parks et al. 2025). Reads mapped to the assembly were calculated by aligning quality-filtered paired-end reads to assembled scaffolds using Bowtie2 (v2.5.4) with default parameters following index construction (Langmead and Salzberg 2012). SAM output files were converted to BAM format using SAMtools (v1.21), and mapping statistics were summarized using the *flagstat* function (Li et al. 2009). The percentage of reads mapping to the assembly was obtained directly from the *flagstat* output as the proportion of mapped reads relative to the total number of reads included in the alignment. In addition, genome-level read recruitment analyses (i.e. assignment of reads to MAGs and unbinned scaffolds) were performed using CoverM (v0.7.0) with the bwa-mem mapper on paired-end reads using default criteria (Aroney et al. 2025), enabling the partitioning of reads into MAG-associated, unbinned, and unassigned fractions. For read partitioning analyses, MAGs were considered on a per-sample basis to ensure consistency with binning outputs (Table S2), whereas pooled MAGs were used separately for cross-site comparative analyses (e.g. ChiPlot construction).

Metagenomic data were further characterized by BLASTx analysis (with an E-value threshold of 1 × 10⁻⁵) to compare the *coxL* gene (form I characterized by the inferred amino acid motif AYXCSFR (Dunfield and King 2004) against our CoxL form I database. Searches were performed against both the recovered MAGs and the unbinned scaffolds from the metagenomic assembly to ensure that genes located outside genome bins were also detected. Additional filtering was performed for the unbinned analysis using the following thresholds: identity ≥60%, alignment length ≥500 amino acids, and E- value ≤ 1 × 10⁻⁵. Further annotation was performed on MAGs containing *coxL* genes in their genome. For this, RAST (Rapid Annotation using Subsystem Technology) was employed for functional annotation using default parameters (Aziz et al. 2008), focusing on CODH-related genes, including both form I and form II. A phylogenetic tree of the MAGs was inferred from the concatenated alignment of 120 bacterial marker genes using GTDB-Tk. The resulting unrooted tree was converted to an iTOL-compatible format for visualization and annotation using the gtdbtk convert_to_itol command (https://ecogenomics.github.io/GTDBTk/index.html), enabling the generation of a chiplot diagram to display phylogenetic relationships among the MAGs. The phylogenetic tree, along with an overall summary of the metagenomic analysis, including *coxL* gene identification, was built using ChiPlot (https://www.chiplot.online; Xie et al. 2023) and the dataset is summarized in Table S3. Metagenomic analyses were performed on a high-performance computing cluster supported by the Research and Specialist Computing Support Service at the University of East Anglia (Norwich, UK).

## Results

### Soil physico-chemical properties

Soil pH was very similar between PDB from the 1401 lava flow and ML from the 1559 lava flow, with averages of 5.2 and 5.3, respectively, while the most recent volcanic deposit (CDL from the 2007 lava flow) had a near-neutral pH of 6.8 (Table S1). The CDL deposit was substantially drier than the other sites with average soil moisture of 5.6%, compared to 59.0% and 64.7% for the 1559 (ML) and 1401 (PDB) samples, respectively, with the highest-moisture soil coinciding with the presence of vegetation (Table S1). Soil temperatures varied inconsistently among sites (20.2°C–28.2°C), likely influenced more by local factors such as elevation and vegetation cover.

### CO uptake by soil microbes

**Figure 2:**
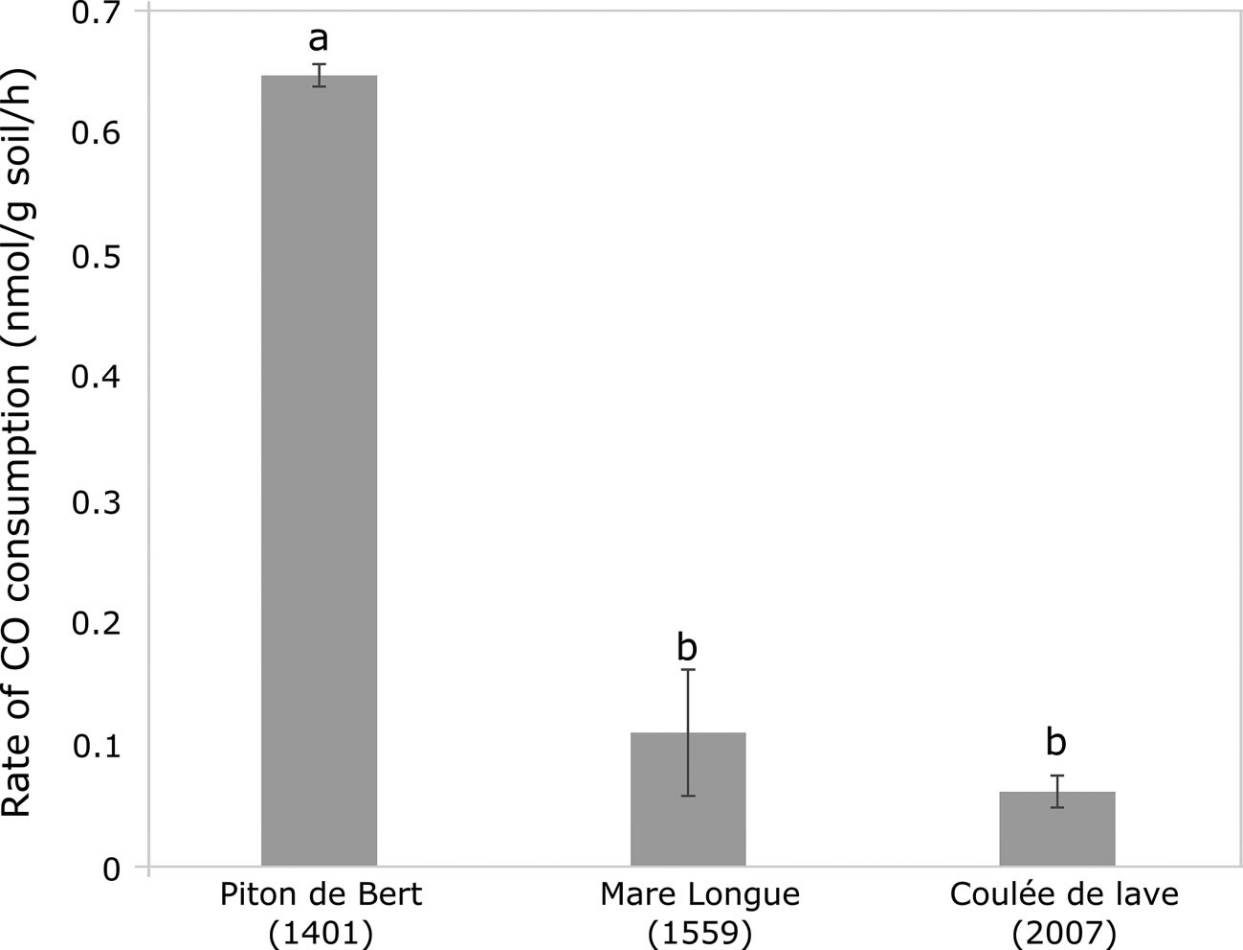
Consumption rates of CO (mean ± SD) in soils from three different locations on Piton de la Fournaise volcano in Réunion Island. Lower-case letters (a and b) denote differences in pairwise comparisons (*P* < 0.05, Tukey’s HSD).

All soil samples exhibited biological consumption of CO (Fig. 2), with no CO consumption observed in autoclaved samples (Fig. S1). CO consumption rates yielded significant variation among the sites (*P* < .01). We observed that the PDB site differed significantly from the other two sample sites (ML and CDL), with a faster rate of CO consumption of 0.63 nmol/h/g soil (Fig. 2). The 1559 (ML) and 2007 (CDL) sites showed lower CO consumption rates and no significant difference between the two with 0.09 nmol/h/g soil and 0.06 nmol/h/g, respectively.

### Total microbial community composition

**Figure 3:**
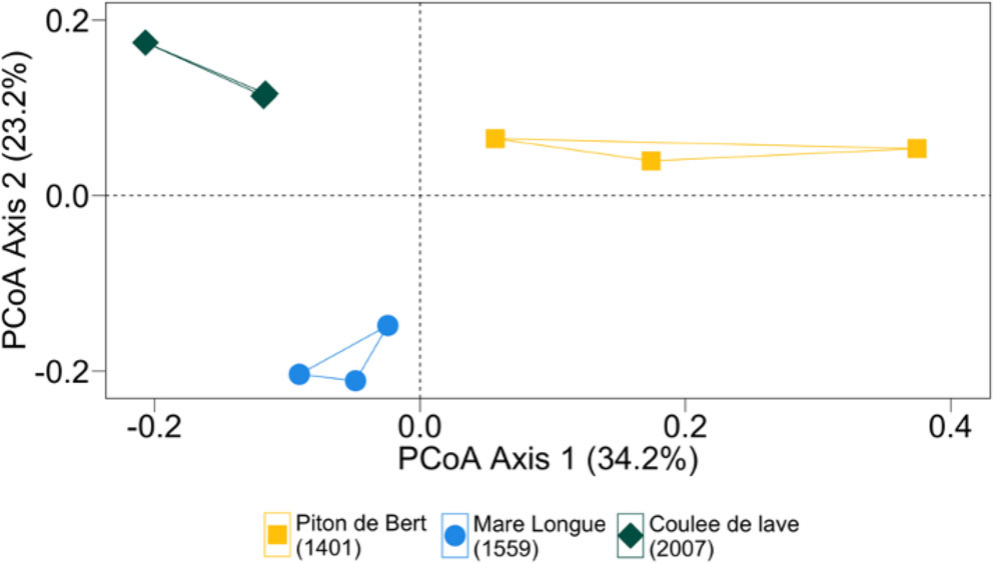
PCoA plots of OTUs (97% sequence similarity) derived from 16S rRNA genes extracted from soil. The legend indicates the eruption date of each sample.

**Figure 4:**
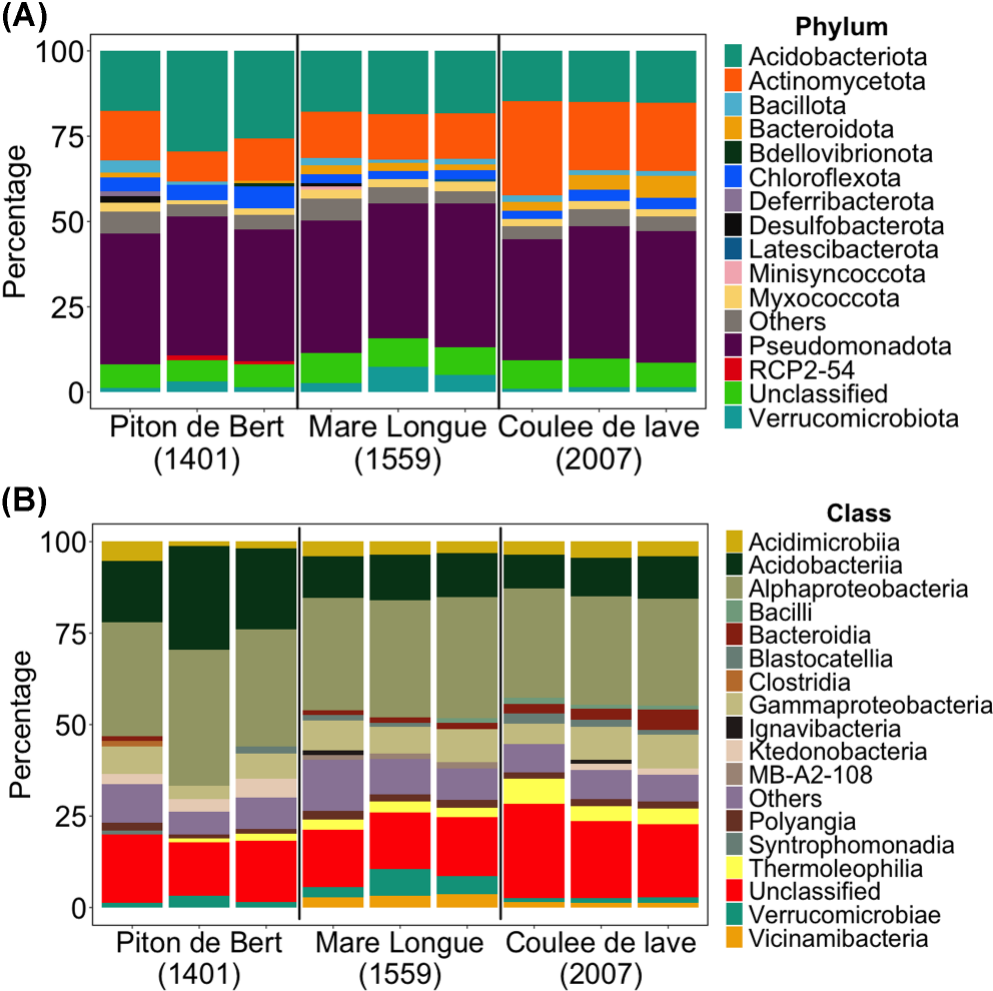
Relative abundance of microbial communities at the phylum (A) and class (B) level based on 16S rRNA genes in the different sample sites. ‘Unclassified’ taxa are those OTUs that were not classified at the genus level. ‘Others’ are those OTUs that were classified but the total abundance was less than 0.8% of all OTUs.

Microbial community composition showed differences in three eruption sites due to separate clusters for all sampling sites (Fig. 3). The variation in community composition is explained by the soil-age (CCA: *F*_1,7_=1.72, *P* = 0.005). For clarity, only the first two PCoA axes were used, which together explained ∼60% of the variation in microbial community composition (Fig. 3, Fig S2). Several notable patterns emerged in the relative abundance at the phylum level between across three sample sites (Fig. 4A). The relative abundance of Acidobacteriota was 10.78% in the 2007 eruption sites (CDL), 13.88% in the 1559 eruption sites (ML), and 20.13% in the 1401 eruption sites (PDB). Actinomycetota varied across sites, with a relative abundance of 17.04% in the 2007 eruption sites (CDL), 10.42% in the 1559 eruption sites (ML), and 9.90% in the 1401 eruption sites (PDB). Bacteroidota, remained relatively low overall, with 3.01% in the 2007 eruption sites (CDL) and 0.35% in the 1401 eruption sites (PDB). In contrast, Pseudomonadota remained relatively stable, maintaining its dominance with percentages around 28%–29% across all three sample sites. Chloroflexota represented 2.35% in the 2007 eruption site (CDL), 4.25% in the 1559 eruption site (ML), and 4.34% in the 1401 eruption site (PDB). Bacillota remained constantly low, at 1.42% in the 2007 eruption sites (CDL) and 1.08% in the 1401 eruption sites (PDB). Finally, Verrucomicrobiota showed variation across sites, with 1.22% in the 2007 eruption sites (CDL), 4.25% in the 1559 eruption sites (ML), and 2.61% in the 1401 eruption sites (PDB).

The composition of bacterial classes across the sample sites also displays variations (Fig. 4B). In the 2007 eruption sites (CDL), Alphaproteobacteria dominated at 29.62%, followed by unidentified Actinobacteria at 13.19% and Acidobacteriia at 10.80%. In the 1559 eruption sites (ML), Alphaproteobacteria remained the most abundant class (31.60%), with Acidobacteriia at 11.59%. In the 1401 eruption sites (PDB), Alphaproteobacteria again dominated (33.05%), accompanied by higher representation of Acidobacteriia (22.40%). Additionally, there was a notable emergence of Ktenobacteria at 5.52%, which was not present in significant proportions in the 2007 (CDL) and 1559 (ML) eruption sites (Fig. 4B).

**Figure 5:**
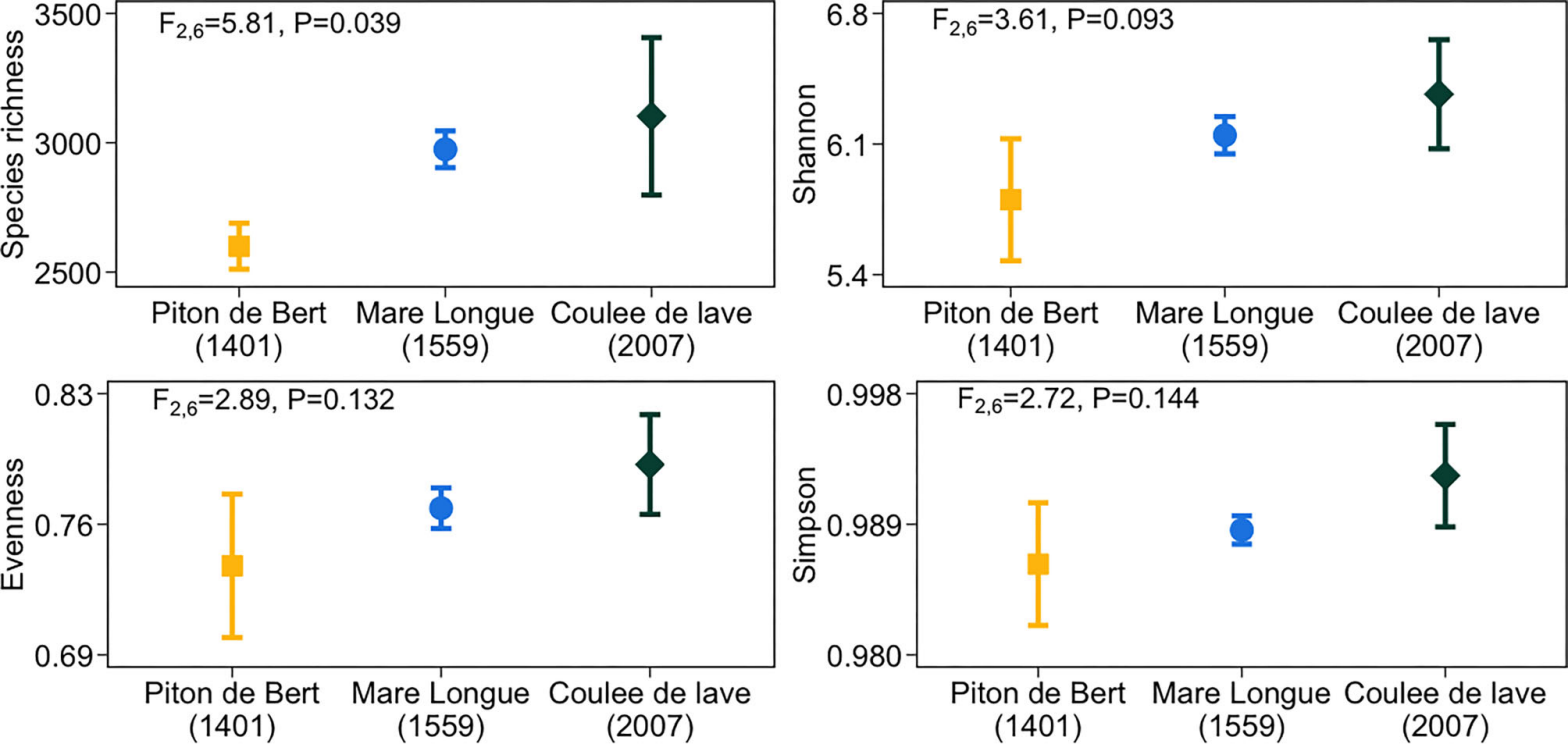
Alpha-diversity of microbes in soils from three different lava flows on Piton de la Fournaise volcano in Réunion Island. Alpha-diversity is evaluated as species richness, Shannon index, species evenness, and Simpson’s diversity. *F*- and *P*-value correspond to those obtained from a one-way ANOVA test performed on the linear model, where the soil-age (three levels) is the explanatory variable and the alpha-diversity measures are the dependent variables. Data are presented as mean values ± standard deviation (n=3).

While all three sites exhibit high species diversity and evenness, there are discernible differences between them (Fig. 5). Species richness and Shannon diversity were comparable between the 2007 (CDL) and 1559 (ML) eruption sites, whereas the 1401 (PDB) was lower, indicating a less diverse microbial community. Nevertheless, all three sites showed high Simpson indices, suggesting a similar level of species evenness across the sampled microbial communities.

### Metagenomic analysis

A total of ~13–15 million scaffold reads were recovered from the soil metagenomes for each site (Table S2). Even though all the sites underwent similar sequencing efforts, the CDL soil (from the 2007 lava flow) had the highest quality assembly, recovering a higher quantity of large scaffolds (308 753 scaffolds >1 kb) compared to the other sites (139 139 scaffolds >1 kb in the ML site, lava 1559, and 279 277 scaffolds >1 kb in the PDB site, lava 1401). Across all samples, a substantial proportion of quality-filtered reads mapped to the assembled scaffolds, with mapping rates of 64.73%, 56.14%, and 69.87% for PDB, ML, and CDL, respectively (Table S2). Genome-resolved read recruitment indicated that only a subset of these reads could be assigned to MAGs, with 3.8%, 5.7%, and 29.8% of reads assigned to MAGs in PDB, ML, and CDL, respectively. Partitioning of reads using different binning approaches revealed substantial variation in the fraction of reads associated with unbinned scaffolds. Using CONCOCT, 11.3%, 8.3%, and 10.8% of reads were assigned to unbinned scaffolds in PDB, ML, and CDL, respectively, with the remaining reads classified as unassigned (84.9%, 86.0%, and 59.4% for PDB, ML, and CDL, respectively). In contrast, MetaBAT2 assigned a larger fraction of reads to unbinned scaffolds (23.9%, 17.9%, and 28.8% for PDB, ML, and CDL, respectively), resulting in lower proportions of unassigned reads (72.3%, 76.4%, and 41.4% for PDB, ML, and CDL, respectively).

**Figure 6:**
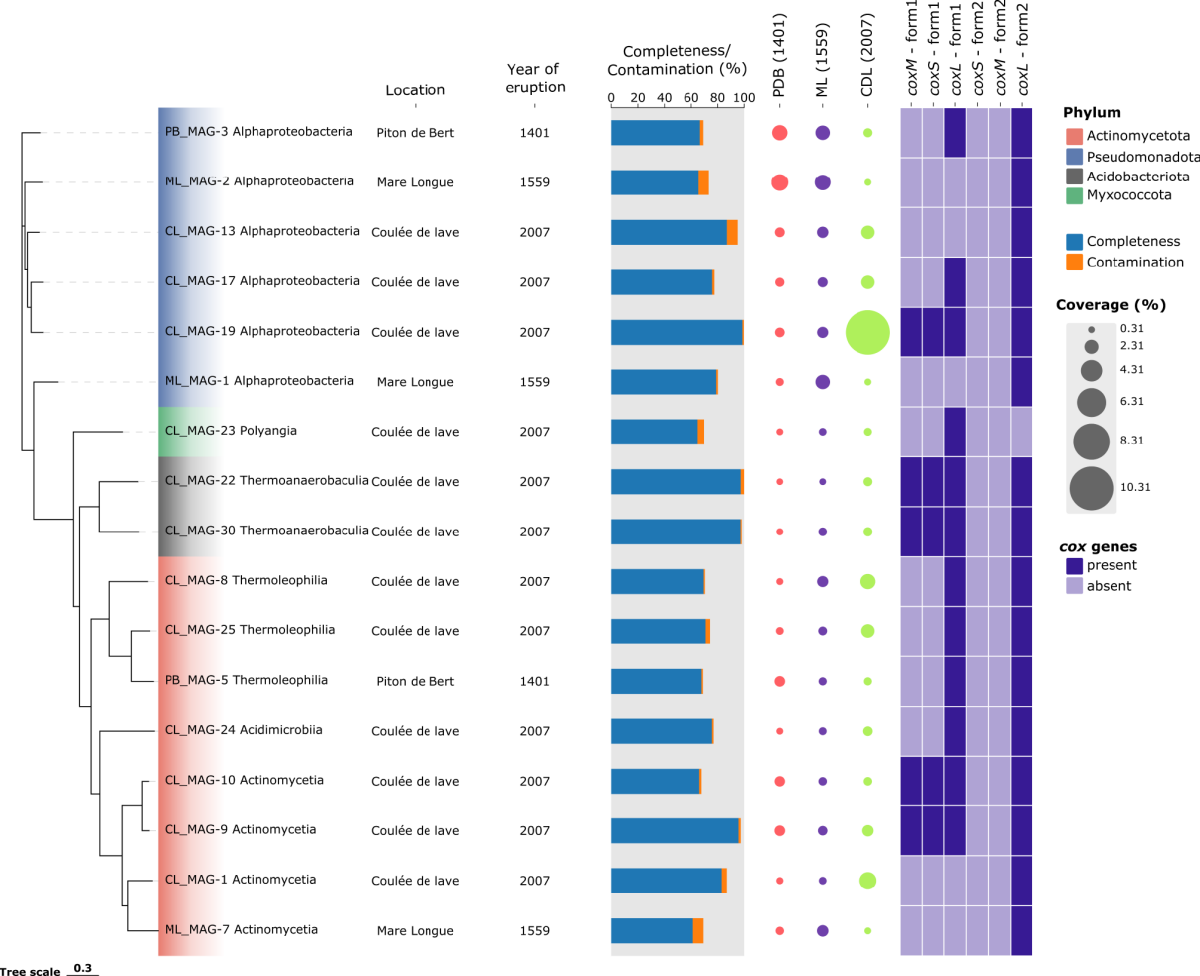
Analysis of MAGs retrieved from soils of different ages from Piton de la Fournaise volcano, classified according to completeness and contamination, coverage (relative abundance), and the presence/absence of CODH-encoding genes.

46 MAGs were retrieved in total. For our study, we analysed only MAGs containing at least one copy of *coxL* gene, totaling 17 MAGs (Table S3). Nine out of the 12 MAGs recovered from the 2007 lava samples (CDL) had >70% completeness and <5% contamination, compared to three MAGs from the 1559 soil (ML) samples and two MAGs from the 1401 samples (PDB), which had >60% completeness and <10% contamination (Table S3). MAGs were affiliated to the phyla Acidobacteriota, Actinomycetota, Myxococcota, and Pseudomonadota (Fig. 6, Table S3). In the PDB site, environmental genomes were retrieved which related to the Pseudomonadota and the Actinomycetota. Environmental genomes from the 1559 soil (ML) were similarly related to the Actinomycetota and Pseudomonadota (Fig. 6). MAGs binned from the CDL site included three assigned to Pseudomonadota (family Xanthobacteraceae), two assigned to the Acidobacteriota, one Myxococcota, and six assigned to Actinomycetota (including the families Gaiellaceae, Ilumatobacteraceae, Solirubrobacteraceae, Streptosporangiaceae, and Nocardioidaceae) (Table S3).

Following BLASTx analysis, these 17 selected MAGs contained >50% matches to *coxL* sequences based on sequence identity, further annotation was performed on these MAGs. RAST was then employed for functional annotation, focusing on CODH-related genes, including both form I and form II. Our results showed that 12 MAGs contained form I *coxL*, with five containing all three structural genes (*coxMSL*). In addition, 16 MAGs contained the putative form II *coxL* gene; however, no associated *coxM* or *coxS* subunits were identified in these genomes. The absence of these additional subunits may reflect incomplete genome recovery, assembly fragmentation, or limitations in binning, which could prevent reconstruction of the full operon. Screening of the unbinned scaffolds (after binning) identified five and two additional *coxL* gene-containing scaffolds in PDB and CDL, respectively, which were not associated with MAGs (Table S4). These unbinned CODH sequences could not be confidently assigned to specific taxonomic groups based on BLAST analysis due to low sequence identity (Table S4). No *coxL*-containing scaffolds were detected in the unbinned fraction of ML (data not shown).

## Discussion

This study aimed to elucidate changes in microbial community composition, complexity, and CO oxidation rates across volcanic deposits of different ages on the Piton De La Fournaise. We identified clear differences in the relative abundance of known CO-oxidizing taxa in different lava flows, with the Pseudomonadota dominating in each site. The Ktedonobacteria, which were previously found to dominate in recent deposits (Hernandez et al. 2020b), were present at low relative abundance in the CDL site and gradually increased with soil age (Fig. S1). Further, the PDB site showed the lowest microbial community diversity (Fig. 5) but the highest rates of CO oxidation (Fig. 2). These observations suggest that CO oxidation remains an active metabolic process in older soils. However, our results do not support the assumption that microbial community complexity increases with soil age. Instead, other environmental factors, such as soil chemistry, elevation, and the availability of plant-derived organic material, are likely stronger drivers of microbial community composition in these environments.

The physico-chemical properties observed in our study revealed notable trends with soil age (Table S1). Specifically, 1401 soil samples exhibited lower pH levels, which could be attributed to the accumulation of organic matter over time. As organic matter decomposes, it generates organic acids that contribute to the acidity of the topsoil. However, additional factors may also play a role. In volcanic environments, acid deposition derived from sulfur dioxide (SO_2_) emissions can lead to the formation of sulfuric acid, which may further contribute to soil acidification (Aiuppa et al. 2007, Oppenheimer et al. 2014). Therefore, the lower pH observed at the 1401 site is likely driven by a combination of soil development processes and volcanic inputs rather than soil age alone. Moreover, our findings indicate that soil moisture content increased with soil age. This may be linked to the enhanced soil structure resulting from the accumulation of organic matter, which allows for better water retention within the soil matrix (Chtouki et al. 2022).

The composition of the PBD and ML sites was distinct from the CDL site, predominantly rocky samples due to the presence of vegetation. This observation suggests a gradual transition to more habitable soil conditions over time, as older samples contained more vegetation material. This pattern could be interpreted as an early indication that biological complexity might increase with soil age, as has been reported in other volcanic systems (Gomez-Alvarez et al. 2007). However, as shown later in our results, this expected trend was not observed in our microbial data, suggesting that the relationship between vegetation development and microbial community change is not straightforward in this system.

The results obtained from the analysis of CO consumption across all different-aged volcanic soils provide compelling support for the hypothesis that CO-oxidizing microbes are active in these soil samples. We observed CO consumption in all samples with no observable lag (Fig. 2) and no abiotic activity (Fig. S1), confirming that consumption is biological in nature. Vegetation is known to influence the abundance and diversity of CO oxidizers, and differences in CO consumption rates may partly reflect the presence of vegetation that produces CO, potentially selecting for microbial communities capable of utilizing this trace gas. These effects likely interact with soil age and ecological succession, which contribute to increased organic matter and microbial communities characteristic of later successional stages, with broader metabolic capabilities (Weber and King 2010). Another explanation could be that higher soil CO₂ concentrations may promote increased CO consumption. For instance, Bénard et al. (2023) found a higher rate of magmatic CO₂ release in soils at higher elevations on the island and that the magnitude of these effects and the type of transfer depended on the geological properties. In volcanic environments, diffuse degassing of reduced gases, including CO, may also exert selective pressure on microbial communities independent of soil age, and could represent an alternative explanation for the observed patterns (Fischer et al. 2019, Rahilly 2020, Fantom et al. 2025).

An alternative explanation for the considerable increase in CO consumption in the PDB site can be seen when comparing with the relative diversity at the class level. The relative abundance of Ktedonobacteria increases in the PBD site (Fig. S3) than in the ML and CDL sites, coinciding with higher rates of carbon monoxide consumption. Members of the class Ktedonobacteria have previously been identified as a lineages with the genetic potential for CO oxidation (King and King 2014, Islam et al. 2019, Hernández et al. 2020a). However, it is important to note that relative abundance in sequencing outputs does not directly reflect metabolic activity, gene expression, or growth rates. Therefore, while the increased representation of Ktedonobacteria in PDB is consistent with a community potentially enriched in taxa capable of CO oxidation, our data do not allow us to infer a direct functional link between abundance and in situ CO oxidation rates. Weber and King (2010) previously correlated greater species richness with greater CO consumption in Hawaiian volcanic deposits, with increased CO oxidation potential coinciding with increased organic carbon and vegetation.

Using 16S rRNA gene sequencing data, our research offers an in-depth analysis of the microbial community structures found at various volcanic eruption sites on Réunion Island. We observed clear spatial segregation of microbial communities (Fig. 3), which corresponds directly to the lava flow’s age, suggesting a distinct community evolution over time. The substantial within-cluster variation in PDB soils could be attributed to the time since eruption. Older soils create a more heterogeneous environment that supports a more diverse, complex, and variable microbial community compared to the younger soils. When using the data to classify organisms, all sites showed communities of bacteria present, confirming that these extreme environments can sustain microbial life. At the phylum and class levels, our data indicate significant variations between sites (Fig. 4). For example, PDB soils showed a greater abundance of Acidobacteriota than in the ML and CDL sites, with a similar rise in Acidobacteriia classes (Fig. 4B). In contrast, Actinomycetota did not exhibit a consistent trend, showing lower relative abundance at the PDB and ML sites. These results suggest that factors other than substrate age may influence the distribution of certain microbial taxa. Overall, the observed patterns suggest a potential association with differences in substrate age; however, additional environmental factors, such as soil chemistry, vegetation cover, and elevation, may also contribute to the variation in microbial community composition across sites. This underscores the dynamic nature of microbial community assembly and ecological succession. Interestingly, Pseudomonadota consistently dominates across all sites, likely due to their capacity for utilizing diverse substrates and rapidly colonizing new areas, playing a crucial role in primary ecological succession (Zhou et al. 2020). Studies have previously found that Pseudomonadota, a group that contains diverse characterized CO-oxidizing bacteria (Santiago et al. 1999, Dawson et al. 2025), are dominant in certain vegetated volcanic deposits ((Weber and King 2010, Hernández et al. 2020b) or unvegetated deposits across diverse environments (Byloos et al. 2018).

When analysing community species richness and evenness, the highest species richness and Shannon diversity indices occur at the 2007 (CDL) site (Fig. 5), contrasting with the hypothesis that microbial community complexity increases with soil age. This suggests that these sites experience rapid colonization by diverse species immediately following an eruption, with drivers of diversity such as associated vegetation potentially playing a greater role than the age of each deposit. Over time, as ecological succession progresses, the community composition becomes more evenly structured, as evidenced by high Simpson indices at all sites. This is evident at the class level, where there is a notable decline of various classes such as Ignavibacteria and Bacilli in the older sites (Fig. 4B), which is likely due to competition from other classes that benefit more from the shift in available nutrients provided by nutrient cycling due to this diverse initial microbial colonization. For example, Ignavibacteria, which comprise facultative or strictly anaerobic microorganisms and have previously been found in microbial mats (Iino et al. 2010), might be more prevalent in newer volcanic soils before aerobic microbes like Alphaproteobacteria establish dominance. Additionally, the presence of Bdellovibrionota in the PDB sample site is interesting due to it having been found previously to be an obligate predator, which can consume other Gram-negative bacteria. It is unclear how this group could influence microbial diversity in these environments, as it was previously associated with both declining (Li et al. 2021) and increased (Johnke et al. 2020) diversity. Our findings are consistent with previous studies, including those from the Kilauea Volcano in Hawaii, which report similar dominant phyla across different volcanic sites (Gomez-Alvarez et al. 2007). However, other global volcanic studies, including Llaima Volcano in Chile, often find younger soils with lower diversity indices and fewer species, which tend to increase as the soil matures and stabilizes due to complex ecological interactions (King et al. 2003b, Gomez-Alvarez et al. 2007, Hernández et al. 2020b). Our results, particularly the high species richness in the CDL site, differ from these findings, suggesting that initial colonization dynamics may also be influenced by site-specific factors, such as elevation in addition to soil age. Díaz et al. (2022) found that elevation significantly influence Simpson indices along an altitudinal gradient on Sumaco Volcano, with the highest values reported at lower elevations. Although all our sampling sites are classified as ‘low elevation’ (Table S1) given that they are located below 2900 m above sea level (Díaz et al. 2022), suggesting a limited influence of elevation on microbial community composition and diversity. Nevertheless, Chloroflexota abundance increased with elevation on both Sumaco and Réunion Volcanoes. However, Díaz et al. (2022) reported that microbial community composition and diversity were more strongly influenced by factors, such as cation exchange capacity and the presence of divalent cations and phosphorus, all of which were correlated with lower elevations.

Our study provides evidence supporting the hypothesis that microbes capable of oxidizing CO are present in volcanic soils; however, contrary to our hypothesis, CO consumption activity was highest at the PDB site. Bacterial phyla such as the alphaproteobacteria, which dominates all soil sample sites, have been previously identified to contain the CODH-encoding gene cluster (Schübel et al. 1995, King 2003b, Dunfield et al. 2004, Hernández et al. 2020a). Additionally, MAGs which contained genes required to express CODH (*coxMSL*) were exclusively isolated from the CDL site (Fig. 6). Ktedonobacteria, which has had extensive research into its CODH-encoding gene cluster through metagenomic studies and isolate-based studies (King and King 2014, Islam et al. 2019, Hernández et al. 2020a), showed an increase in relative abundance in the PDB sample compared to the two other soil sites (Fig. S3). This suggests that CO-oxidizing bacteria persist across different stages of soil restoration, including conditions where organic carbon content is not as limited, indicating that CO oxidation may contribute to their ecological succession in these soils. A study of microbial respiration in soils on Kilauea Volcano (King 2003a) also suggested that CO oxidation was selected for by microbial communities even when heterotrophic metabolism was possible.

On average, between ∼56% and 70% of the quality-filtered reads mapped back to assembled scaffolds (Table S2), indicating that a substantial proportion of the metagenomic data is represented in the assemblies. However, genome-resolved analyses showed that only a relatively small fraction of reads could be assigned to MAGs, particularly in PDB and ML (3.8% and 5.7%, respectively), with a higher proportion observed in CDL (29.8%), demonstrating that the genome-resolved fraction represents only part of the total microbial community. This indicates that a large portion of the assembled-metagenomic signal is not captured within reconstructed genomes and remains unresolved at the genome level. This is consistent with previous studies on complex soil ecosystems. Soil metagenomes are known to capture only a fraction of the underlying genomic, metabolic, and phylogenetic diversity, with much of the microbial community remaining unsampled or unassembled (Howe et al. 2014, Anthony et al. 2024). For example, Howe et al. (2014) showed that their sequenced soil metagenomes yield assemblies representing only ∼10% of the total sequence data, despite recovering millions of predicted genes and a whole range of functional diversity. These findings highlight a fundamental limitation of genome-resolved metagenomics in highly complex environments such as soils, where both spatial heterogeneity and temporal dynamics further complicate comprehensive community reconstruction. Consequently, the MAGs recovered in this study should be interpreted as representing only a partial view of the microbial community and its functional potential.

In the exploration of soil metagenomes across different eruption ages, the recovery of MAGs demonstrated a notable disparity influenced by soil age. The most comprehensive assembly was achieved in the 2007 site (CDL), which boasted the highest number of large scaffolds, suggesting robust assembly quality. The more effective binning of genomic data in the 2007 site could be attributed to less genomic complexity at this stage of soil development, which would be in line with the hypothesis of complexity increasing with age from eruption; however, that challenges the previous results from analysis of 16S rRNA sequencing data. The taxonomic profiling of the MAGs revealed distinct phyla predominance corresponding to soil age. In the 2007 site (CDL), one quarter of MAGs were affiliated with the Pseudomonadota, including families known for their versatile metabolic capabilities, which may confer advantages in early soil colonization stages (Dawson et al. 2025). Two MAGs recovered from the middle-aged soil (ML site) were also affiliated with the Pseudomonadota, while the third was affiliated with the Actinomycetota, which is known to rely more on degrading complex organic compounds, potentially reflecting a shift in the organic composition of the soil as it matures. The PDB site, similarly, yielded two MAGs related to Pseudomonadota and the Actinomycetota (Fig. 6), which may similarly reflect a shift to more metabolically versatile microorganisms. Gene-centric analysis of assembled scaffolds showed that *coxL* genes were present both in MAGs and unbinned contigs, suggesting that CO oxidation potential may also be associated with organisms not recovered as MAGs.

## Conclusions

In conclusion, while our study did not fully support the initial hypothesis regarding an increase in microbial community complexity with increasing soil age, it did enhance our understanding of the role of CO-oxidizing microbes in volcanic soils. We observed particularly high species richness in the CDL soils, suggesting that early colonization is shaped not only by soil age but also by site-specific factors, such as elevation. Overall, our research contributes to the growing evidence that volcanic eruption sites serve as unique natural laboratories for examining microbial colonization and succession. The high-diversity phase observed immediately following an eruption, coupled with subsequent microbial community stabilization, highlights the complex and dynamic nature of these ecosystems. Our results also indicate that the observed patterns likely reflect a combination of space-for-time substitution and local environmental variables, rather than a fully controlled chronosequence. These findings emphasize the need for further longitudinal and biogeochemical studies to fully understand the environmental factors and temporal dynamics influencing microbial communities in volcanic landscapes, which will enhance our predictions of ecological recovery and inform conservation strategies in extreme environments.

## Supplementary Material

fiag062_Supplemental_Files

## Data Availability

Sequence data were deposited in the NCBI Sequence Read Archive (SRA) under the bioproject accession numbers: PRJNA1367882 for amplicon-sequencing data and PRJNA1367899 for metagenome- assembled genomes (Table S3) and raw data.
