# Supplementary material for: Carbon monoxide oxidizers in soils of different ages from Piton de la Fournaise volcano
Source: FEMS Microbiol Ecol. 2026 Jun 9;102(7):fiag062. doi: 10.1093/femsec/fiag062 (PMC13285871; doi:10.1093/femsec/fiag062)
Supplement: fiag062_Supplemental_Files [file fiag062_supplemental_files.zip › Wildbur-SI-final.pdf]

Supplementary Information for:

Carbon monoxide oxidizers in soils of different ages from Piton de la Fournaise volcano

Constance Wildbur[1], Robin A. Dawson[1], Shamik Roy[1,2], Claudine Ah-Peng[3,4], Mikk Espenberg[5], Marcela Hernández[1*]

[1] School of Biological Sciences, University of East Anglia, Norwich, NR4 7TJ, UK

[2] Chair for Forest Zoology, Technische Universität Dresden, Tharandt 01737, Germany

[3] UMR PVBMT, Université de la Réunion, 97410, Saint-Pierre, La Réunion, France

[4] OSU-Réunion, Université de la Réunion, 97400, Saint-Denis, La Réunion, France

[5] Department of Geography, Institute of Ecology & Earth Sciences, University of Tartu, Tartu, Estonia

*Corresponding author: Marcela Hernández, School of Biological Sciences, University of East Anglia, Norwich, NR4 7TJ, UK, marcela.hernandez@uea.ac.uk

Supplementary Annex

Table S1
Table S2
Figure S1
Figure S2
Figure S3

Supplementary Annex

Statistical analyses and OTU classification – R script

```
library(vegan)
reu_melt_wide=read.csv.csv("~/Library/Mobile Documents/com~apple~CloudDocs/UEA/connie CO paper/reunion_volcano_full_data.csv", head=T)
reu_melt_wide1=reu_melt_wide[,c(1:10)]

### calculate alpha diversity
#Species richness
reu_melt_wide1$spec_rich <- specnumber(reu_melt_wide[,-c(1:10)])

#Shannon
reu_melt_wide1$shannon <- diversity(reu_melt_wide[,-c(1:10)], index="shannon")

#Simpson
reu_melt_wide1$simpson <- diversity(reu_melt_wide[,-c(1:10)], index="simpson")

#evenness
reu_melt_wide1$evenness <- reu_melt_wide1$shannon/log(reu_melt_wide1$spec_rich)

mod_alpha <- (lm(alpha-diversity-measures ~ eruption,
        data = reu_melt_wide1, na.action = na.omit))
#Note: eruption denotes the numerical value of the year of volcanic eruption

anova(mod_alpha)


### community composition visualization with PCoA

dist_reu<- vegdist((decostand(reu_melt_wide[,-c(1:10)], method= 'hellinger')),method='bray', na.rm = T)
pcoa_reu <- pcoa(dist_reu)

### statistical test to test whether soil age is an important factor in the separation of the groups seen in PCoA plots
vare.cca=cca((decostand(reu_melt_wide[,-c(1:10)],
          method= 'hellinger')) ~ eruption, data=reu_melt_wide)
#Note: eruption denotes the numerical value of the year of volcanic eruption
anova(vare.cca)
```

Table S1. Location and physico-chemical properties of the soil samples

Site	Coordinates	Eruption year / (elevation - masl)	Average pH	Average soil moisture (%)	Soil temp (°C)	Air temp (°C)	Soil observations
Piton de Bert (PDB)	21.2788831 S, 55.6980607 E	1401 / (2242)	5.2±0.3	64.7	28.2	33.7	Damp, no rocks, some vegetation
Mare Longue (ML)	21.3512651 S, 55.7392276 E	1559 / (282)	5.3±0.1	59.0	20.2	21.3	Dry, lots of rocks
Coulée de Lave (CDL)	21.2866284 S, 55.7957900 E	2007 / (124)	6.8±0.3	5.6	25.1	29.5	Dry, lots of rocks

Table S2. Summary of metagenome assembly statistics and read recruitment across sampling locations. Reads mapped to the assembly (%) were calculated by aligning quality-filtered paired-end reads to assembled scaffolds using Bowtie2 (v2.5.4) and summarising alignments with SAMtools (v1.21) using the *flagstat* function. Genome-level read recruitment was performed using CoverM (v0.7.0, relative abundance, (%)). Reads were assigned to (i) scaffolds incorporated into MAGs and (ii) scaffolds not assigned to any MAG (unbinned scaffolds) for each sample and binning approach (CONCOCT and MetaBAT2). Reads not recruited to either category were classified as unassigned. All percentages are calculated relative to the total number of post-quality-filtered reads per sample. MAGs were derived from a consensus binning approach (BinRef) integrating outputs from multiple binning tools; therefore, MAG-associated read recruitment values are shared across binning partitions, whereas unbinned scaffold fractions are specific to each binning method. For each binning approach, values represent an independent partitioning of the metagenomic dataset (MAG-associated + unbinned + unassigned = 100%) and are not directly comparable across binning methods.

Location	Piton de Bert (PDB)	Mare Longue (ML)	Coulée de lave (CDL)
**Assembly-level mapping**			
Total reads (post-QC)	197,152,272	154,516,628	199,102,956
Number of scaffolds	15,189,418	13,321,547	13,143,294
Reads mapped to assembly (%)	64.73	56.14	69.87
**CONCOCT-based partitioning**			
Reads assigned to MAGs (%)	3.8	5.7	29.8
Reads assigned to unbinned scaffolds (%)	11.3	8.3	10.8
Unassigned reads (%)	84.9	86.0	59.4
**MetaBAT2-based partitioning**			
Reads assigned to MAGs (%)	3.8	5.7	29.8
Reads assigned to unbinned scaffolds (%)	23.9	17.9	28.8
Unassigned reads (%)	72.3	76.4	41.4

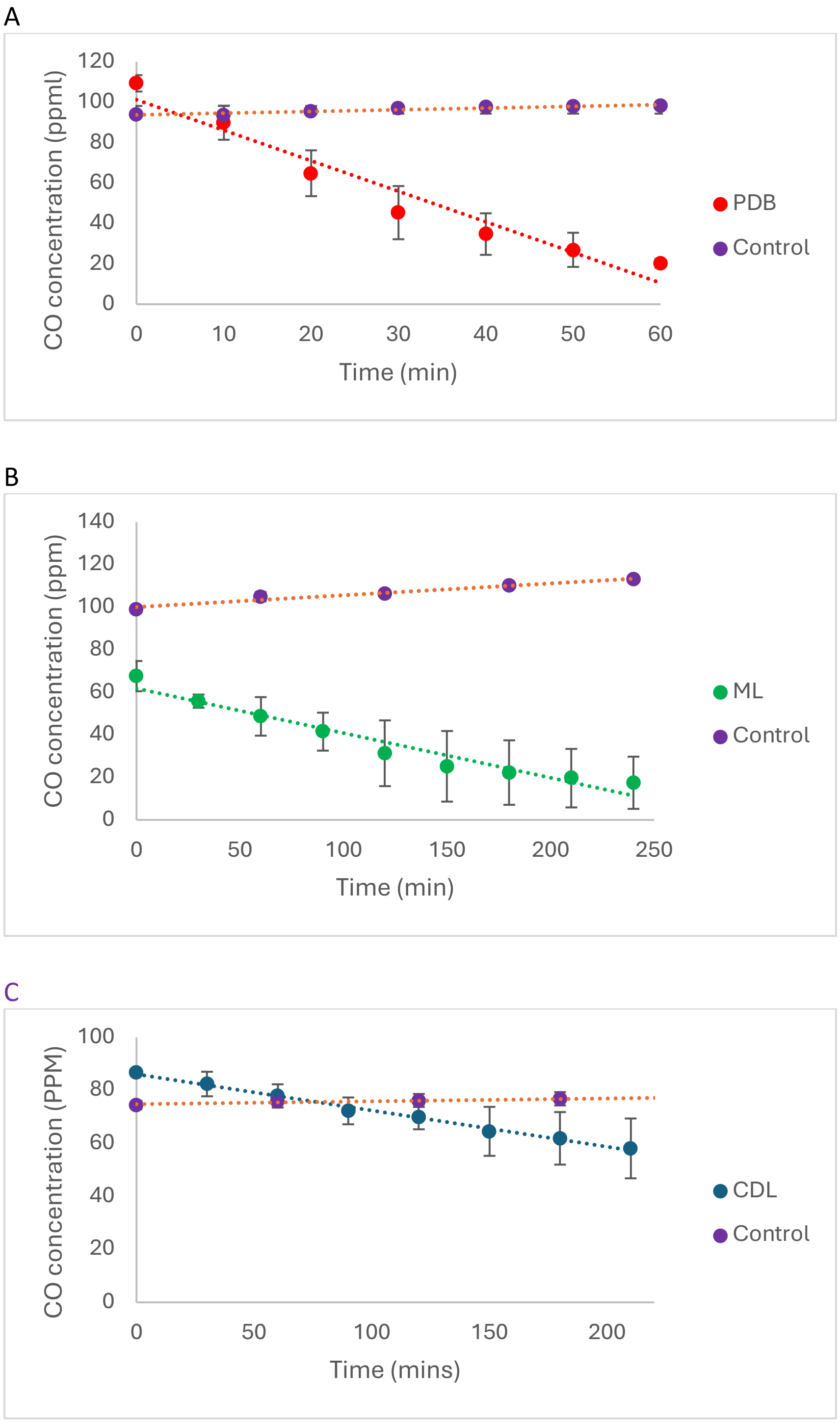

Figure S1. Rate of CO consumption by Piton de Bert (PDB) (A), Mare Longue (ML) (B) and Coulée de lave (CDL) (C) soil samples compared to controls from autoclaves soil samples. Points represent mean values with standard deviations of independent triplicate incubations.

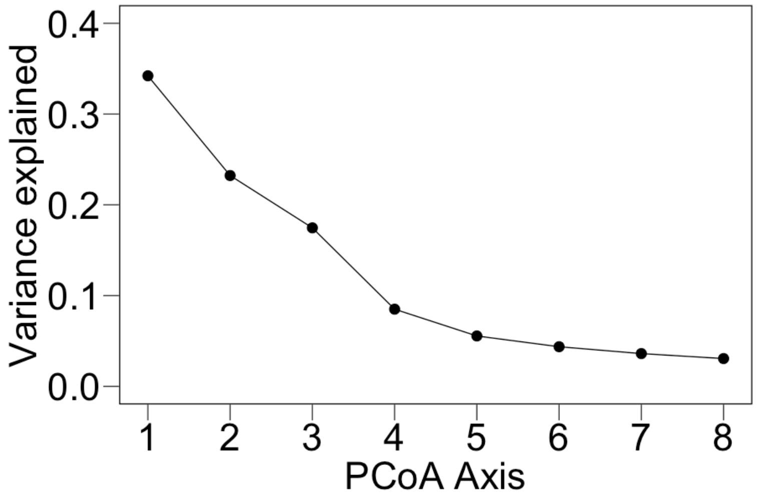

Figure S2. Scree plot showing the variance explained by each principal coordinate (PCoA) axis. The first axis explains the highest proportion of variance, with subsequent axes contributing progressively less, indicating the relative importance of each axis in capturing the dataset's multivariate structure.

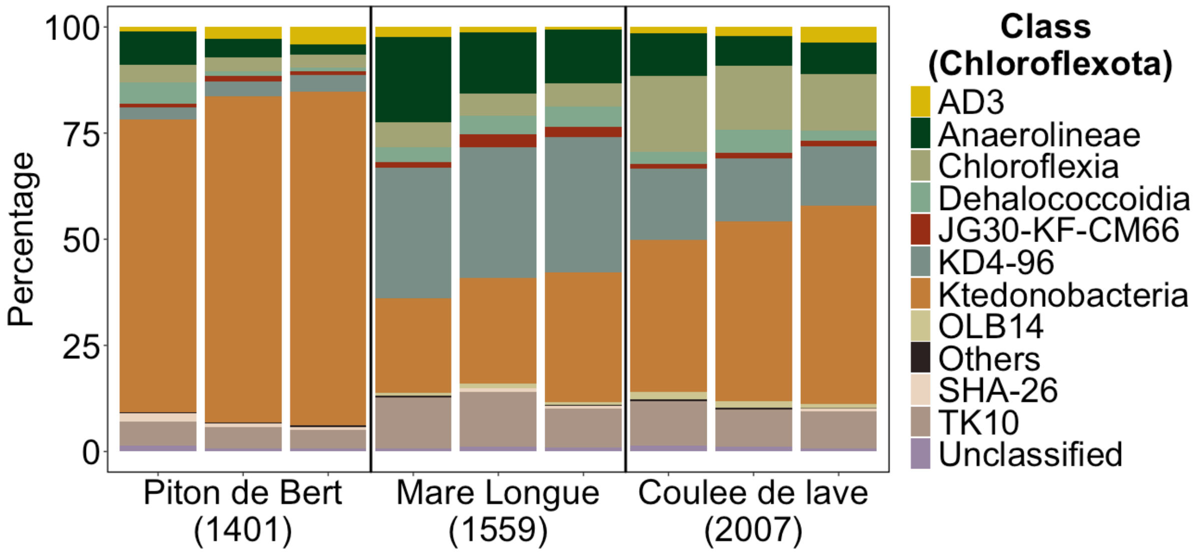

Figure S3. Relative abundance of microbial communities at the Chloroflexota class level based on 16S rRNA genes in the different sample sites. “Unclassified” taxa are those OTUs that were not classified at the genus level. “Others” are those OTUs that were classified but the total abundance was less than 0.5% of all OTUs.
